# Salivary Creatinine Estimation as an Alternative to Serum Creatinine in Chronic Kidney Disease Patients

**DOI:** 10.1155/2014/742724

**Published:** 2014-04-10

**Authors:** Ramesh Venkatapathy, Vasupradha Govindarajan, Nirima Oza, Sreejith Parameswaran, Balamurali Pennagaram Dhanasekaran, Karthikshree V. Prashad

**Affiliations:** ^1^Department of Oral Pathology & Microbiology, Mahatma Gandhi Post Graduate Institute of Dental Sciences, Gorimedu, Puducherry 605006, India; ^2^Department of Nephrology, Jawaharlal Institute of Postgraduate Medical Education and Research, Gorimedu, Puducherry 605006, India

## Abstract

*Context*. Sampling blood for serum analysis is an invasive procedure. A noninvasive alternative would be beneficial to patients and health care professionals. * Aim*. To correlate serum and salivary creatinine levels and evaluate the role of saliva as a noninvasive alternative to serum for creatinine estimation in chronic kidney disease patients.* Study Design*. Case-control study. * Methods*. Blood and saliva samples were collected from 37 healthy individuals and 105 chronic kidney disease patients. Serum and salivary creatinine levels were estimated using automatic analyser. * Statistical Analysis*. The serum and salivary creatinine levels between controls and cases were compared using *t*-test. Correlation between serum and salivary creatinine was obtained in controls and cases using Pearson correlation coefficient. Receiver operating characteristic analysis was done to assess the diagnostic performance of salivary creatinine. Cut-off values were established for salivary creatinine. * Results*. Serum and salivary creatinine levels were significantly higher in CKD patients than controls. The correlation was negative in controls and positive in cases. Area under the curve for salivary creatinine was found to be 0.967. A cut-off value of 0.2 mg/dL gave a sensitivity of 97.1% and specificity of 86.5%. * Conclusion*. Saliva can be used as a noninvasive alternative to serum for creatinine estimation.

## 1. Introduction

Chronic kidney disease (CKD) is progressive reduction in renal function. The prevalence and incidence are increasing worldwide with diabetes and hypertension as the leading cause [1]. This condition requires frequent serum analysis to diagnose and monitor therapeutic outcomes and to ascertain prognosis. Creatinine, a waste product of muscle metabolism, is primarily excreted by kidneys and its level in serum is used as an index to renal function [2].

Collection of blood for serum analysis is an invasive procedure causing anxiety and discomfort to the patients. Certain amount of blood loss is associated with each dialysis procedure in CKD patients which amounts to about 4 to 20 mL, with an additional loss which results from frequent blood sampling [3]. Also the patients undergoing dialysis are at greater risk of developing Hepatitis B and C [4, 5], potentially increasing the risk of health care professional to blood borne diseases. Thus, a simple diagnostic test that provides a reliable evaluation of disease status and stages would be of value to both the clinicians and the patients.

Saliva, a multiconstituent biologic fluid secreted by the salivary glands, is the major contributor of oral health. It has a cutting edge over serum because saliva collection is a noninvasive, simple, and economic procedure that can be performed by the patient with minimal involvement from medical personnel. When required a repeat sample can be easily obtained and is suitable for all age groups. It also provides a cost-effective approach for the screening of large populations. Saliva as a diagnostic medium will also be a boon to patients suffering from clotting disorders like haemophilia and in patients with compromised venous access [6–10].

There are several preliminary studies with promising results which show that saliva can be used to detect lung cancer, pancreatic cancer, breast cancer, and type II diabetes. But to bring it to a clinical reality we need further scientific validation for each disease and also required to benchmark the diagnostic capacity of saliva against other bodily fluids [11–13].

With this background a study was planned to determine the diagnostic ability of saliva as an alternative to blood to estimate creatinine in chronic kidney disease patients.

## 2. Aims and Objectives

The aims of the study are to correlate the serum and salivary creatinine levels and to evaluate the role of saliva as a noninvasive alternative to serum for creatinine estimation in chronic kidney disease patients.

## 3. Materials and Methods

Ethical clearance was obtained from institutes' ethical committee to perform this study. The study population comprised 105 patients already diagnosed with chronic kidney disease (CKD) and 37 healthy volunteers (age and gender matched) who had no complaint or major illness in recent past were selected as controls.

Based on the GFR estimated using Cockcroft Gault formula, the CKD patients were found to be in stage 4 and stage 5 chronic kidney disease. The patients were either under medical management alone or were also undergoing hemodialysis/peritoneal dialysis.

After obtaining a written informed consent, a clinical examination of the oral cavity was performed and the case details were recorded on a special proforma. Blood and whole unstimulated saliva samples were obtained.

All the samples were collected between 9:00 and 11:00 a.m. to minimize the effect of diurnal variation. In patients undergoing hemodialysis, the sample was collected prior to dialysis. Two mL of blood was drawn from antecubital vein with minimal trauma under aseptic condition. Two mL of whole saliva was obtained under restful conditions, in a sterile graduated container by spitting method. The participants were instructed to refrain from eating and drinking at least 90 min before collection and thoroughly rinse mouth with deionised/distilled water prior to the collection to void the mouth of saliva. They were asked to sit in a comfortable position with eyes open and head tilted slightly forward and to avoid swallowing and oral movements during collection and to pool the saliva in the floor of the mouth and spit every 60 seconds or when they experience an urge to swallow the fluid accumulated. This was done until 2 mL of whole saliva was obtained [14, 15].

All collected samples were centrifuged at 3000 RPM for 10 minutes [15]. Salivary supernatant and serum were separated. The samples were assayed immediately in automatic analyser (EM360 chemistry analyser with ISE module) using creatinine estimation kit (Swemed diagnostics) by Jaffe kinetic reaction [16].

The data obtained were entered in the MS excel sheet and data analysis was done using SPSS v17.0. Pearson's correlation coefficient was used to test the correlation between serum and salivary creatinine levels. Linear regression equations were derived to estimate the serum level from the salivary creatinine level. ROC analysis was performed to find whether salivary creatinine levels can distinguish the diseased subjects (cases) from nondiseased (controls). The overall performance was determined by the area under the curve. The cut-off value was determined based on the best trade-off between the sensitivity and specificity.

## 4. Results

The study population comprised a total of 142 individuals among which 105 patients were suffering from CKD and 37 were healthy volunteers.

### 4.1. Gender- and Age-Wise Distribution of Cases and Controls

Group 1 comprised 37 healthy volunteers as controls. There were 21 males and 16 females. The mean age of the controls was 44.5 years with a standard deviation of 14.97. The minimum age was 19.0 and the maximum was 68.0 years.

Group 2 comprised 105 CKD patients. There were 84 males and 21 females. The mean age of this group (cases) was 47.5 years with a standard deviation of 15.2. Their ages ranged between 19.0 and 70.0 years. Based on their estimated GFR, 38 patients were classified into stage 4 CKD (GFR: 15–30 mL/min) and 67 patients into stage 5 CKD (GFR: <15 mL/min).

Among the 67 stage 5 CKD patients, 22 were undergoing hemodialysis and 10 were undergoing peritoneal dialysis along with medical management. Remainders of the patients were only under medical management without dialysis.

Vast majority of the patients being referred to nephrology department were in late stages of CKD and the consecutive patients selected in our study happened to be in stage 4 and stage 5.

In controls the serum creatinine values ranged between 0.6 and 1.4 mg/dL with a mean of 0.89 mg/dL (SD 0.168) and the salivary values ranged between 0.1 and 0.3 mg/dL with a mean of 0.12 mg/dL (SD 0.06).

In CKD patients the serum creatinine level ranged between 2.0 and 13.8 mg/dL with a mean of 5.96 mg/dL (SD 3.048) and range of the salivary creatinine level was found to be 0.2–2.3 mg/dL with a mean of 0.66 mg/dL (SD 0.485).

To see if the salivary values were also elevated in CKD patients like the serum values, we did a comparative study. The mean serum and the salivary creatinine concentration were found to be significantly higher in CKD patients compared to controls (Table 1).

To know if there was any association between serum and salivary creatinine and if changes in serum creatinine are accompanied by changes in salivary creatinine, we performed a correlation analysis of both study groups (cases and controls). The correlation between serum and salivary creatinine in controls was found to be negative, *r* = −0.326, and in CKD patients a significant positive correlation was found, *r* = 0.731 (Table 2).

**Figure 1:**
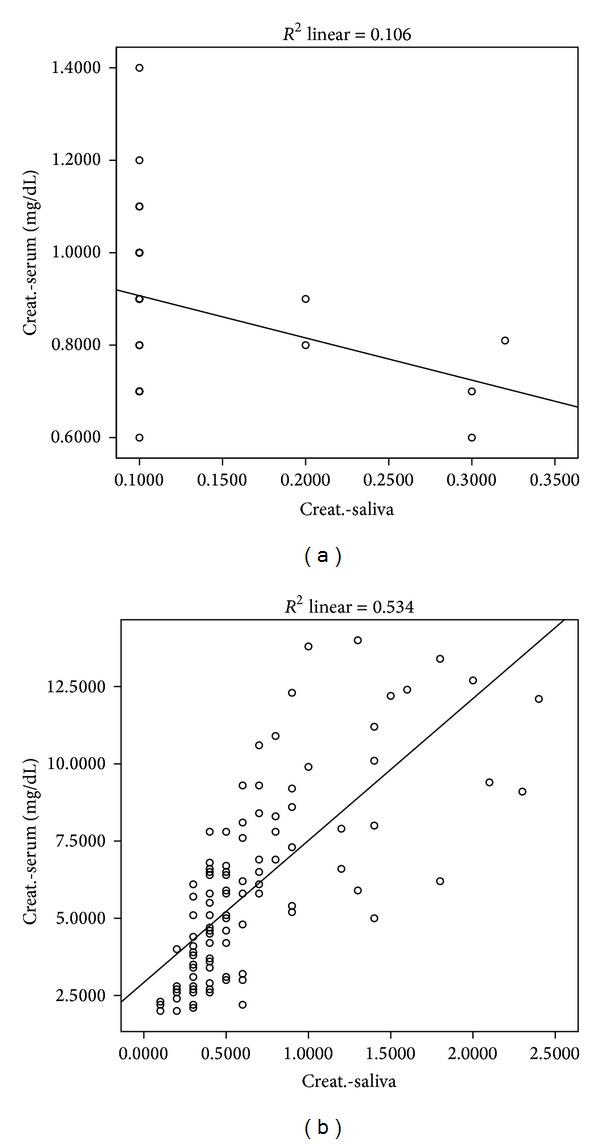
(a) Scatter diagram showing linear correlation between salivary serum and creatinine levels among controls. (b) Scatter diagram showing linear correlation between salivary serum and creatinine levels among CKD patients.

**Figure 2:**
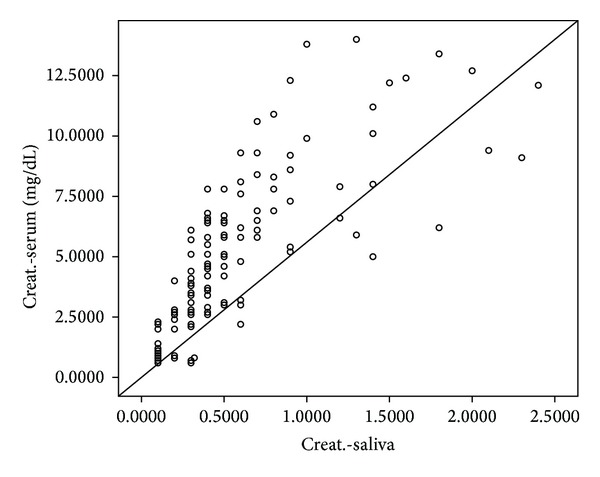
Scatter plot showing serum and salivary levels including both cases and controls.

Linear regression equation was performed to estimate serum creatinine levels from the salivary creatinine value (Table 3). The linear regression equation* Y* = 0.998 + (−0.913) × (Salivary Cr) was obtained in control (Figure 1(a)). The linear regression equation* Y* = 2.924 + (4.595) × (Salivary Cr) was obtained in CKD patients (Figure 1(b)). The linear regression equation with 2/3 of the study subjects, with the other 1/3 used as a validation population, is* Y* = 1.846 + 5.748 (Salivary Cr) (Figure 2).

**Figure 3:**
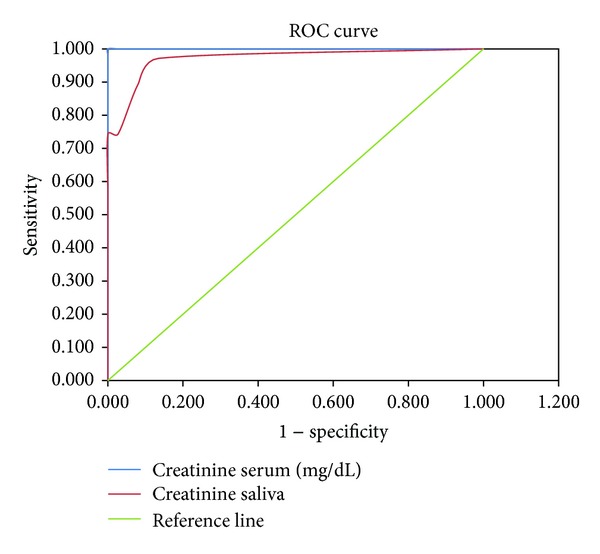
Receiver operating curve for serum and salivary creatinine levels.

To assess the diagnostic potential of saliva when compared to serum creatinine, that is, to separate the group being tested into those with and without the disease in question, ROC analysis was performed (Figure 3). The total area under the curve obtained was 1.000 for serum creatinine and 0.967 for salivary creatinine (Table 4).

Sensitivity and specificity for different values of salivary creatinine were established and a cut-off value of 0.2 mg/dL was determined as this gave a best trade-off with sensitivity of 97.14% and specificity of 86.5%. The other cut-off values with the sensitivity and specificity are mentioned in Table 5.

## 5. Discussion

Creatinine is a waste product of metabolism that is primarily excreted by kidneys. Virtually all the creatinine that is filtered at the glomerulus is excreted without reabsorption in the tubules and so its level in the blood is used as an index to renal function [2]. The normal range of serum creatinine is 0.6–1.5 mg/dL [2] and salivary creatinine is 0.05–0.2 mg/dL [17]. The ranges obtained in our control group were in accordance with this.

We observed a significantly high creatinine level both in serum and saliva of CKD patients compared with controls (Table 1). Similar observation was made by Xia et al. [18] and Davidovich et al. [19]. This is because the kidneys are unable to excrete creatinine in renal failure and hence its concentration in blood increases [2]. The increased concentration in saliva may be because of increased serum creatinine which creates an increased concentration gradient which in turn increases the diffusion of creatinine from serum to saliva in CKD patients [20]. It is also possible that saliva may be an attempted alternative route of excretion by the body in a compromised renal function state.

To know if there was any association between serum and salivary creatinine and if changes in serum creatinine are accompanied by changes in salivary creatinine, we performed a correlation analysis of both study groups (CKD patients and controls) and found a negative correlation in controls and a positive correlation in CKD patients (Table 2). Similar observation was made by Lloyd et al. [21]. This may be because of the following reason.

Creatinine is a large molecule, with high molecular weight (MW 113 Da and molecular radius of 3.2 Å) maintained at constant plasma levels by kidneys. They also exhibit low lipid solubility. Thus in a healthy state under normal conditions owing to its physical properties it is unable to diffuse easily across the cells and the tight intercellular junction of the salivary gland [2, 16, 22, 23]. Hence, a low negative correlation was obtained in controls. But in the diseased state possibly there is an alteration in the permeability of the salivary gland cells [24]. Also the increased serum creatinine levels in CKD patients create a concentration gradient that facilitates increased diffusion of creatinine from serum in to saliva [20]. So, a good positive correlation was obtained in CKD patients.

But in contrast to our study, Xia et al. [18] found a positive correlation in both their cases (*r* = 0.971) and controls (*r* = 0.932) while we obtained a positive correlation only in CKD patients similar to Lloyd et al. [21].

To know the functional relationship between serum and salivary creatinine and to estimate the serum creatinine from salivary creatinine values, we did a linear regression analysis and regression equation was derived for both the controls and the CKD patients (Table 3).

Before a salivary diagnostic test can replace a more conventional one, the diagnostic value of a new salivary test has to be compared with accepted diagnostic methods. The accuracy of this new test depends on how well it separates the group being tested into those with and without the disease in question [25]. Sensitivity and specificity are the basic measures of the accuracy of a diagnostic test. Hence, to ascertain the diagnostic potential of saliva as an alternative, ROC analysis was performed (Figure 3).

Accuracy is measured by the area under the ROC curve. The higher area under the curve (0.967) obtained in our study for salivary creatinine suggests that salivary creatinine is a good alternative diagnostic test to discriminate CKD patients from healthy individuals. Similar large area under the curve 0.897 was obtained by Xia et al. [18].

In this study, various cut-off points for salivary creatinine to diagnose renal disease were obtained using ROC analysis considering serum creatinine as gold standard. A cut-off value of 0.2 mg/dL gave a sensitivity of 97.14% and specificity of 86.5% (Table 5). That is, people with salivary creatinine values above 0.2 mg/dL are more likely to suffer from CKD and must be subjected for further medical evaluation for appropriate management.

Thus the results of the present study suggest that the saliva can be used as alternative diagnostic medium for estimating serum creatinine in chronic kidney disease patients.

## 6. Summary and Conclusion

This study was an effort to harness the advantage of saliva as a noninvasive diagnostic fluid in chronic kidney disease patients, which has the potential to dramatically reduce anxiety and discomfort associated with blood sampling procedures and also increases their willingness to undergo frequent health inspections that will greatly increase the opportunity to monitor their general health over time and to diagnose morbidities in the early stage. Sampling saliva instead of blood is suitable for all age groups and also reduces the occupational risks to laboratory personnel.

The satisfactory result obtained in our study suggests that saliva can be used as a noninvasive diagnostic tool for estimating serum creatinine in chronic kidney disease patients. However, this study is not without limitations. The study group consisted only of stage 4 and stage 5 CKD patients (though not intentional). To authentically say that saliva can be used to diagnose CKD, a study comprising patients in all the stages of CKD and healthy controls should be performed, thus laying the foundation for further research. This study leaves us with a promise that saliva has the potential to be used as an alternative to serum.

## Figures and Tables

**Table 1:** Comparison of serum and salivary creatinine levels between CKD patients and controls using *t* test.

	Group	*N*	Mean	Std. deviation	Std. error mean	*t*-value	*P*-value
Serum (mg/dL)	Controls	37	0.887	0.168	0.028	16.969	<0.001**
	Cases	105	5.956	3.048	0.297		

Saliva (mg/dL)	Controls	37	0.122	0.060	0.010	11.126	<0.001**
	Cases	105	0.660	0.485	0.047		

**Denotes statistically highly significant.

**Table 2:** Table showing correlation between serum and salivary creatinine in control and CKD patients using Pearson correlation.

	*r*	*P*
Controls	−0.326	0.000**
Cases	0.731	0.049*

*Correlation significant at 0.05 level (2-tailed); **Correlation significant at 0.01 level (2-tailed).

**Table 3:** Table showing linear regression analysis of serum and salivary creatinine for controls and CKD patients.

Group	Linear regression equation	Sig.	*R* ^2^
Control	*Y* = 0.998 + (−0.913) × (Salivary Cr)	0.049	0.106
Cases	*Y* = 2.924 + (4.595) × (Salivary Cr)	<0.001	0.534
Combined population	*Y* = 1.846 + (5.748) × (Salivary Cr)	<0.001	0.643

**Table 4:** Table showing area under the ROC curve.

Test result Variable(s)	Area	Standard error	Asymptotic sig.	Asymptotic 95% confidence interval	
				Lower bound	Upper bound
Serum (mg/dL)	1.000	0.000	<0.001**	1.000	1.000
Saliva (mg/dL)	0.967	0.014	<0.001**	0.940	0.995

**Statistically highly significant.

**Table 5:** Sensitivity and specificity analysis of salivary creatinine for different cut-off values considering serum creatinine as the gold standard.

Salivary creatinine (mg/dL)	Sensitivity (%)	Specificity (%)
0.1	100.00	0.00
**0.2**	**97.14**	**86.5**
0.3	89.52	91.89
0.4	74.29	100
